# Editorial: Standardizing cognitive endophenotype profiling in bipolar disorder and schizophrenia

**DOI:** 10.3389/fpsyg.2025.1606517

**Published:** 2025-04-30

**Authors:** Pau Soldevila-Matías, Renato de Filippis, Joan Vicent Sánchez-Ortí, Inmaculada Fuentes-Durá, Rosa Ayesa-Arriola, Patricia Correa-Ghisays

**Affiliations:** ^1^Center for Biomedical Research in Mental Health Network (CIBERSAM), Health Institute, Carlos III, Madrid, Spain; ^2^INCLIVA - Health Research Institute, Valencia, Spain; ^3^Faculty of Psychology and Speech and Language Therapy, University of Valencia, Valencia, Spain; ^4^Psychiatry Unit, Department of Health Sciences, University Magna Graecia of Catanzaro, Catanzaro, Italy; ^5^Valdecilla Biomedical Research Institute (IDIVAL), Santander, Spain

**Keywords:** bipolar disorder, clinical psychiatry, cognitive impairment, endophenotype, mental health, neurocognition, neurosciences, schizophrenia

The study of cognitive endophenotypes has gained prominence in psychiatric research as a means to elucidate the underlying mechanisms of severe mental illnesses such as bipolar disorder (BD) and schizophrenia (SCZ) (Luperdi et al., 2019; Soldevila-Matías et al., 2022). These endophenotypes, encompassing deficits in executive function, attention, and memory, have been consistently observed in both affected individuals and their first-degree relatives, supporting their heritability and potential role in early risk detection (Bora et al., 2008; Gur et al., 2006). However, a significant challenge in the field remains the lack of consensus on standardized criteria for defining these cognitive markers, leading to discrepancies across studies (Toulopoulou et al., 2010). Recent advancements, such as the MICEmi framework (Correa-Ghisays et al., 2022), offer structured methodologies to enhance consistency and reliability in cognitive endophenotype profiling. Standardization in this domain has the potential to refine psychiatric nosology, improve early identification of at-risk individuals, and inform targeted interventions (Insel and Cuthbert, 2015). Further research is necessary to validate these approaches across diverse populations and determine their predictive value for disease onset and progression.

This Research Topic was developed to address the pressing need for standardization in cognitive endophenotype research for mental illnesses such as BD and SCZ. By promoting the adoption of structured methodologies like MICEmi, this Research Topic seeks to consolidate previous findings, reduce methodological variability, and enhance the reproducibility of results. Through this initiative, we aim to advance the integration of cognitive endophenotypes into both clinical practice and research frameworks, ultimately improving diagnostic accuracy, refining classification systems, and informing personalized treatment strategies.

The study conducted by Oscoz-Irurozqui et al. explored the link between cannabis use and genetic variability in endocannabinoid receptors in patients suffering from first episode psychosis (FEP). Authors examined 50 FEP patients of European ancestry (mean age 26.14 years, 76% male), classified as cannabis users (58%) or non-users. Two Single Nucleotide Polymorphisms (SNPs) were analyzed: CNR1 rs1049353 and CNR2 rs2501431.

According to their results, cannabis users showed a trend toward more severe positive psychotic symptoms and better manipulative abilities. Moreover, carriers of the T allele in CNR1 rs1049353 had higher disorganization scores. The cognitive benefits of cannabis use on manipulative abilities were modified by CNR2 rs2501431, with G allele carriers performing better than AA carriers, while the opposite was seen in non-users. Although preliminary, these results suggest that CNR1 and CNR2 genetic variants influence symptom severity and cognitive performance in FEP, interacting with cannabis use (Oscoz-Irurozqui et al.).

The paper published by Liao et al. investigated the effects of comorbid alexithymia on cognitive impairment in chronic schizophrenia, through a cross-sectional analysis of 695 patients with schizophrenia (464 males, 231 females), and assessing alexithymia using the Toronto Alexithymia Scale (TAS-20), cognitive function with the Repeatable Battery for the Assessment of Neuropsychological Status (RBANS), and psychiatric symptoms via the Positive and Negative Syndrome Scale (PANSS). Authors found a prevalence of 31.4% of patients having comorbid alexithymia, with a higher occurrence in males. Still, patients with alexithymia had significantly higher PANSS negative symptom and total scores. Moreover, patients suffering from alexithymia showed greater deficits in immediate memory, delayed memory, and language, reflected in lower RBANS scores. Therefore, regression analysis identified alexithymia as a predictor of language deficits and overall lower cognitive performance in schizophrenia.

Authors concluded that patients with schizophrenia and comorbid alexithymia experience worse cognitive function than those without. Alexithymia, along with certain demographic factors, may contribute to cognitive impairment in chronic schizophrenia (Liao et al.).

In the third article included in our Research Topic, the group lead by Wang et al. analyzed the role of negative cognitive biases in depression and their influencing factors, including subtype, age, gender, age of onset, family history, and education level. According to the reported results, depression was linked to increased negative attention, memory, interpretation, and rumination biases. Negative rumination bias was more pronounced in the melancholic subgroup than in the anxious subgroup. In addition, correlation analysis showed that negative rumination bias was associated with family history and age of onset. These findings enhance understanding of cognitive biases in depression and suggest that rumination-focused therapies should be tailored differently for melancholic and anxious depression. This could aid in developing personalized treatment approaches for major depressive disorder (Wang et al.).

Finally, Giralt-López et al. examined whether Theory of Mind (ToM) deficits are trait- or state-dependent by analyzing the influence of clinical vulnerability markers (basic symptoms and psychotic-like experiences) in unaffected siblings of individuals with schizophrenia. They enrolled 65 individuals (38 schizophrenia patients, 27 healthy siblings), and administrated the Hinting Task (HT) to explore the ToM impairment, the Frankfurt Complaint Questionnaire (FCQ) for basic symptoms, the Community Assessment of Psychic Experiences (CAPE) for psychotic-like experiences, and the Family Interview for Genetic Studies for family history. They found that patients had significantly lower ToM scores than their siblings, but this difference became non-significant after adjusting for clinical vulnerability markers. Among healthy siblings, higher depressive symptoms (FCQ) and negative psychotic-like experiences (CAPE) were associated with poorer ToM performance. These results suggest that ToM deficits are not exclusive to SZ but also linked to clinical vulnerability factors. ToM could serve as an endophenotypic marker, helping identify individuals at higher risk due to genetic predisposition (Giralt-López et al.).

The standardization of cognitive endophenotype profiling represents a critical step forward in psychiatric research, particularly for BD and SCZ. By addressing the methodological inconsistencies that have hindered progress in the field, structured approaches like the ones described in this Research Topic offer a promising avenue for enhancing the reliability and reproducibility of findings. This Research Topic highlights the importance of establishing consensus-driven criteria to improve early risk detection, refine psychiatric classification, and facilitate the development of personalized treatment strategies. Future studies should focus on validating these standardized methodologies across diverse populations and evaluating their predictive utility for disease onset and progression. Ultimately, the integration of standardized cognitive endophenotype profiling into clinical and research settings has the potential to transform our understanding of severe mental illnesses and improve patient outcomes.
